# New York Correspondence

**Published:** 1866-01

**Authors:** 


### NEW YORK CORRESPONDENCE.

Messrs. Editors,—Thinking that some fragmentary correspondence from New York may interest your readers, the following is submitted:

The New York Woman's Hospital, at the corner of Madison avenue and 29th st., is a State institution, which was originated with special reference to the treatment of vesico-vaginal fistula while the discoveries of Dr. J. Marion Sims were new in the minds of the people and of the profession.

Dr. Sims gave the hospital its reputation, and the hospital gave Dr. Sims a field for his operations on patients who could not pay for attendance.

In 1862 Dr. Sims went to Paris to practice the new art, and he is now in London, where his old success is said still to attend him.

Upon the departure of Dr. Sims, the hospital came under the charge of Dr. Thomas Addis Emmet, who had been Dr. Sims' assistant, and whose dexterity and success have kept up the reputation of the institution. About fifty cases of vesico-vaginal fistula are operated upon annually, and about a hundred and fifty other cases of diseases and deformities peculiar to women are operated upon with the skill of an expert, and with proportionate success. Dr. Emmet does not claim the success in ovariotomy boasted of by many operators, he having lost just half his cases.

His operation for procidentia uteri, which is a modification of that of Dr. Sims', is claimed to be free from the defects of all previous operations.

The union of two parallel lines permits the uterus to crowd itself into the narrow canal on one side or the other of the longitudinal septum.

The hour-glass vagina, made by causing the union of a transverse belt of denuded surface, was found to be liable to the difficulty of strangulating the neck of the uterus, and of gradually descending to the os externum, pressed down by the weight above.

Dr. Sims adopted the expedient of peeling off the mucous membrane corresponding with two sides of a triangle, of which the apex is at the lower end of the vagina behind, and the base at the upper. The union of these made a pocket behind, into which it was found the uterus would insinuate itself, even in the retroverted position, making it necessary in extreme cases to slit open this septum and restore the vagina to its former state.

Dr. Emmet's modification consists in dissecting off a belt of mucous membrane across the base of this triangle, so that when the denuded surfaces are made to adhere, the pocket behind becomes a perfectly closed sac, the top of which constitutes a floor upon which the uterus may rest without danger of strangulation.

Dr. Emmet employs the interrupted suture with silver wire for all operations about the genital organs, dispensing with clamps, buttons and quills, thinking there should never be more strain than the suture alone will hold.

Besides the indoor patients, about a thousand outdoor patients are prescribed for annually.

Sims' duck-bill speculum is uniformly employed in vaginal examinations, and to steady the uterus a sharp-pointed tenaculum is thrust into any convenient place about the mouth or neck, and so destitute of tactile or painful sensibility is the part, that the patient rarely feels the puncture.

For exploring the interior of the uterus, Simpson's sound is altogether abandoned, and a copper wire, a little larger than a knitting needle, galvanized, and mounted upon a convenient handle, is employed. This is carefully introduced, having a moderate curve, and as soon as it meets resistance, it is withdrawn, and curved between the fingers according to the supposed direction of the cavity, and the withdrawals, bendings and introductions are repeated until the sound has the curvature which will permit its introduction without meeting resistance, the mouth of the uterus being all the time held in one place by the tenaculum.

A conductor for medication, which may be an untempered watch-spring, mounted upon a convenient handle, is then bent to the same curvature, and wrapped with cotton wool, dipped in a strong tincture of iodine, a solution of chromic acid, of nitrate of silver or other substance, and introduced to the fundus of the uterus. The patient is then required to keep quiet, to avoid the colic which may arise from the sympathy of the intestines with the uterus when the patient is allowed to move about.

Procidentia, with increased weight of the uterus, is treated for the removal of the chronic inflammation before any operation is performed on the vagina.

Dr. Emmet makes the criticism upon the operation for dysmenorrhœa, practiced by Dr. Simpson, of slitting up the lateral parts of the neck, that in the first place, the stricture is higher up, at the vaginal junction with the uterus, and is generally caused by flexure occasioned by the contraction attending unequal distribution of chronic inflammation, with unequal deposition of contractile plastic material. And in the next place, this division permits the anterior and posterior lips to be drawn apart by the traction of the anterior and posterior walls of the vagina in standing, walking and lifting. This keeps up irritation and inflammation, extending higher up, with not only a continuance but an aggravation of the dysmenorrhœa.

Dr. Emmet scarifies and reunites these surfaces, whether they have resulted from accident or design, and thus restores the annular character of the os.

For the reason here explained, his incision, if he makes any, for anteflexion, is anterior, and by an instrument introduced concealed, and cutting out as it is withdrawn. After any incision of the uterus, the vagina is plugged, and the woman left in a horizontal position to avoid hemorrhage, of which it is admitted there is great danger. D. P.

21 W. 12th-st., N.Y., Jan. 12th, 1866.

